# Thin layer spectroelectrochemical (RVC-OTTLE) studies of pertechnetate reduction in acidic media

**DOI:** 10.1007/s10967-014-3016-2

**Published:** 2014-02-16

**Authors:** M. Chotkowski, A. Czerwiński

**Affiliations:** Faculty of Chemistry, University of Warsaw, Pasteura 1, 02-093 Warsaw, Poland

**Keywords:** Pertechnetate, Optically transparent thin layer electrodes

## Abstract

The electroreduction of the pertechnetate ions has been examined in sulfuric acid solutions (0.5–4 M) using optically transparent thin layer spectroelectrochemical (RVC-OTTLE) cell. Soluble Tc(III), TcO^2+^ and [Tc(μ-O)_2_Tc]^3+/4+^ species with absorption bands at 420–450, 400, and 502 nm, respectively, were found to be formed during the reduction of TcO_4_^−^ ions. The strongly acidic medium was found to stabilize technetium ionic forms with lower oxidation states. Spectroelectrochemical measurements performed in 4 M H_2_SO_4_ show different stability of dimeric structure of Tc(III, IV) and simple TcO^2+^ ions. The monooxotechnetium(IV) ions can be electrooxidized at potentials lower than 0.6 V versus Ag,AgCl_(sat.)_ while dimeric structures of Tc, [Tc(μ-O)_2_Tc]^3+/4+^, are electrooxidized to pertechnetate ions at potentials higher than 0.8 V versus Ag,AgCl_(sat.)_

## Introduction

Technetium-99 is one of nuclear fission products with a long half-life time (^99^Tc:* T*_1/2_= 2.11 × 10^5^ years) and a relatively high fission yield (6 %). Tc has a very high mobility in the environment [1]. This element is present at almost every stage of nuclear fuel reprocessing streams due to its high ability to be extracted with uranium by TBP (tri-*n*-butyl phosphate) [2, 3]. Technetium exists in a wide range of oxidation states, therefore, the spectroelectrochemistry is especially useful to study electrochemical and chemical processes with participation of Tc.

Generally, non-complexed technetium compounds can be ordered in the following series, according to their stability in the acidic media, Eq. (1):

1 $$ {\text{Tc}}\left( {\text{VII}} \right){\text{ > Tc(IV) > Tc(III) > Tc}}\left( {\text{VI}} \right) $$

where Tc(VI) compounds are the most unstable ones. Recent studies [4] showed that only in extreme acidic media (13 M H_2_SO_4_) the reduction of pertechnetates can lead to formation of stable TcO^3+^ species. The UV–Vis spectrum of these species is characterized by the bands: the first one at 255 nm and the second one at 650–700 nm, the last one with a small absorptivity (*ε* = 34 M^−1^ cm^−1^). A similar experiment performed in 6 M H_2_SO_4_ did not reveal formation of Tc(V) [4].

It was described in the literature that technetium(IV or III/IV) ionic species can exist in the acidic media in various forms, as e.g. dimeric structures of Tc, [Tc(μ-O)_2_Tc]^3+/4+^ [5, 6] or polyoxopolymetallic Tc_3_O_4_^4+^ species [7]. These species can be characterized spectroscopically by a broad Vis band with a maximum absorption near 500 nm. TcO^2+^ oxocations can be characterized spectroscopically by a band at 400 nm [8].

Despite mixed electrochemical and chemical pathways of pertechnetate ions reduction, the technetium compounds are good subjects for spectroelectrochemical studies [9, 10]. However, only in a few cases the spectroelectrochemical studies were performed in aqueous solutions [11–13]. The experiments performed by Paquette and Lawrence [11] showed that in slightly basic media (pH 8) Tc(IV) and Tc(III) are stabilized by bicarbonates. These species were spectroscopically detected by weak bands in Vis range at 512 for Tc(IV), 470 and 630 nm for Tc(III). Thin layer spectroelectrochemical techniques have been also employed by Huber et al. [12] to study the behaviour of TcCl_6_^2−^ and TcBr_6_^2−^ in concentrated acidic media. Our previous study [13] performed in 0.5–4 M H_2_SO_4_ in a gold-optically transparent cell (Au-OTE) indicated that dimeric structures of Tc(III,IV) are generated during electroreduction of pertechnetate ions.

The pertechnetate ions generate two absorption bands in the UV–Vis range: at 244 and 288 nm [14]. Therefore, the UV–Vis spectroscopy is especially suitable for studying the reactions involving these ions. The spectroelectrochemistry is particularly useful [9] for determining the mechanism of electrochemical reduction processes of technetium(VII). The working electrode is usually manufactured from an optically transparent material [10, 15], e.g. from RVC (Reticulated Vitreous Carbon), which was applied by Norwell and Mamantov [16] and Marassi et al. [17–19] in an optically transparent thin layer cell (OTTL-RVC cell). It has been demonstrated that for model systems, like o-tolidine or K_3_[Fe(CN)_6_], the shape of UV–Vis spectroscopic signal recorded in an optically transparent cell is similar to the shape of electrochemical voltammetric current versus potential (*j* vs.* E*), curve [9, 15, 17]. The most convenient form of presentation of spectroscopic data recorded in OTTLE-type cells is a plot showing a time derivative of the absorbance (d*A*/d*t*) as a function of the electrode potential. Such a plot, i.e. voltabsorbommogram, can be considered as an equivalent of simultaneously recorded current versus potential curve. However, such voltabsorbommogram can be calculated only for a single wavelength which has to be selected as the representative one for the analysed compound. Therefore, only one UV–Vis band is used for calculation of cyclic voltabsorbommograms although UV–Vis spectra can correspond to more than one electronic transition. The decrease of the absorbance for selected wavelength causes appearance of "negative" wave on cyclic voltabsorbommograns (for this wavelength).

The purpose of this work is to investigate the spectroelectrochemical behaviour of pertechnetate ions in 0.5–4 M sulphuric acid. It will be shown that the use of the OTTLE-RVC technique in conjunction with cyclic voltammetry allows for the determination of the mechanism related to the generation and stability of technetium species detected electrochemically on the electrode and spectroscopically in the solution.

## Experimental

Spectroelectrochemical measurements were carried out in a home made optically transparent thin layer cell. A reticulated vitreous carbon—RVC (thickness 2 mm; 100 ppi porosity, ERG Aerospace Corporation) was used as a working electrode, and a platinized platinum gauze, as a counter electrode [20]. A saturated Ag/AgCl electrode was used as a reference electrode, and all the potentials in the text are referred to this electrode.

All measurements were performed at 298 K, the solutions were deoxygenated with Ar (4 N). The solutions were prepared using high purity distilled water (Millipore^®^) and high purity chemicals: potassium pertechnetate, K^99^TcO4 (obtained from Forschungszentrum Dresden—Rossendorf—Institute of Radiopharmacy) and H_2_SO_4_, (POCh, Poland). The electrochemical and spectroscopic measurements were performed simultaneously using CHI604 (CH Instruments) electrochemical analyzer and MultiSpec 1500 (Shimadzu) spectrophotometer, respectively.

## Results and discussion

**Fig. 1:**
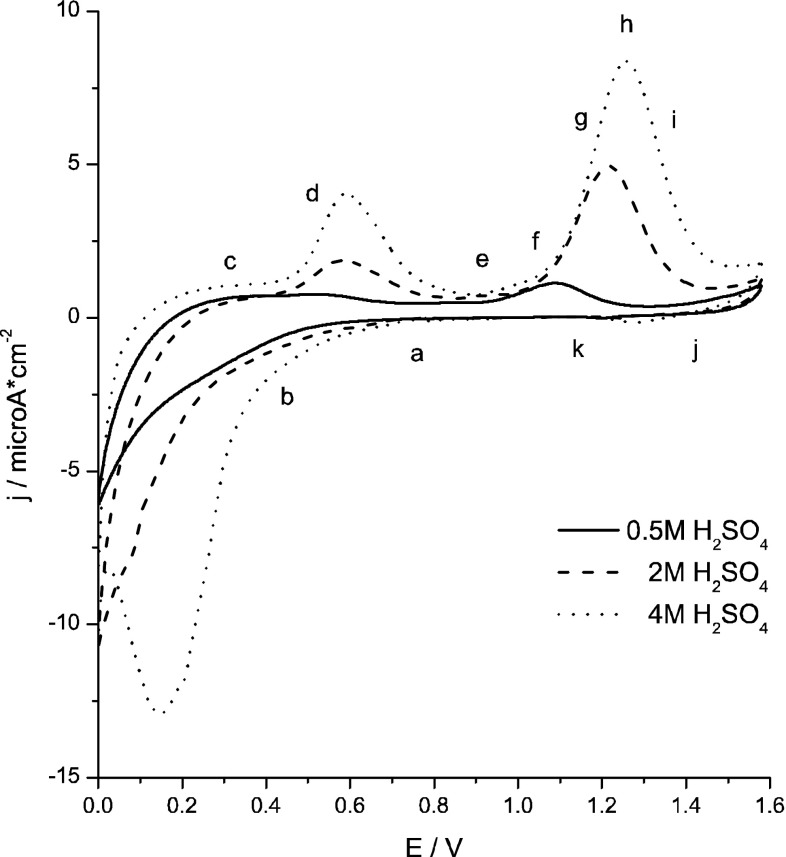
Cyclic voltammograms recorded in 0.5 mM KTcO4 at various concentration of H_2_SO_4_,* v* = 2 mV/s, *E*_start_ = 0.6 V

Figure 1 presents cyclic voltammograms recorded at scan rate of 2 mV/s for a RVC-OTTL electrode in 0.5, 2 and 4 M H_2_SO_4_ with the addition of 0.5 mM KTcO_4_. The reduction of pertechnetate ions in 0.5 M H_2_SO_4_ results in a weak increase of cathodic current at potentials lower than 0.4 V. According to the literature data [21] this electrochemical signal can be attributed to the electroreduction of pertechnetate ions to TcO_2_·*x*H_2_O for solutions with pH greater than 3. Grassi et al. [22] postulated that electroreduction of pertechnetate ions in H_2_SO_4_ with the concentration as high as 0.5 M H_2_SO_4_ generates not only Tc(IV) but also Tc(III) species. Other reports [5] indicated Tc_*n*_O_*y*_^(4*n*−2*y*)+^ polymeric species as the product of pertechnetates electroreduction. Also Maslennikov et al. [23] reported that Tc(VII) can be reduced to Tc(III) species in nitric acid solutions. Vichot et al. [7] suggested that the discussed process can be accompanied by synproportionation of electrogenerated Tc(III) species and Tc(VII) ions to Tc(IV). In more concentrated sulfuric acid solutions, the electoreduction wave is better developed which suggests changes both in the mechanism of TcO_4_^−^ ions electroreduction and in composition of the resulting products.

Presence of two anodic waves observed on cyclic voltammograms at potentials of 0.4 and 0.9–1 V indicates that pathways of electrooxidation of low valence technetium species to TcO_4_^−^ cannot be treated as a process composed with the same steps as electroreduction of pertechnetate ions and appearing in a reversed order. The results presented in this work and in our previous paper [13] devoted to studied on electrochemistry of pertechnetates with the use of gold electrodes in the same concentrations of sulphuric acid solutions strongly indicate that electrooxidation of Tc(III) species to Tc(IV) is not the only one process that contributes to formation of the broad and poor shaped first anodic wave observed in the potential range of 0.2–0.6 V. Thus, an additional electrochemical process, e.g. electrooxidation of Tc(IV) to Tc(VII) species, must occur in the same potential region. According to the literature [24] the Tc(III) species are very prone to oxidation to Tc(IV). The second anodic wave appears at much higher potentials (0.8–1.2 V). At such high potentials other Tc(III, IV) species are electrooxidised to pertechnetate ions as it will be described later.

**Fig. 2:**
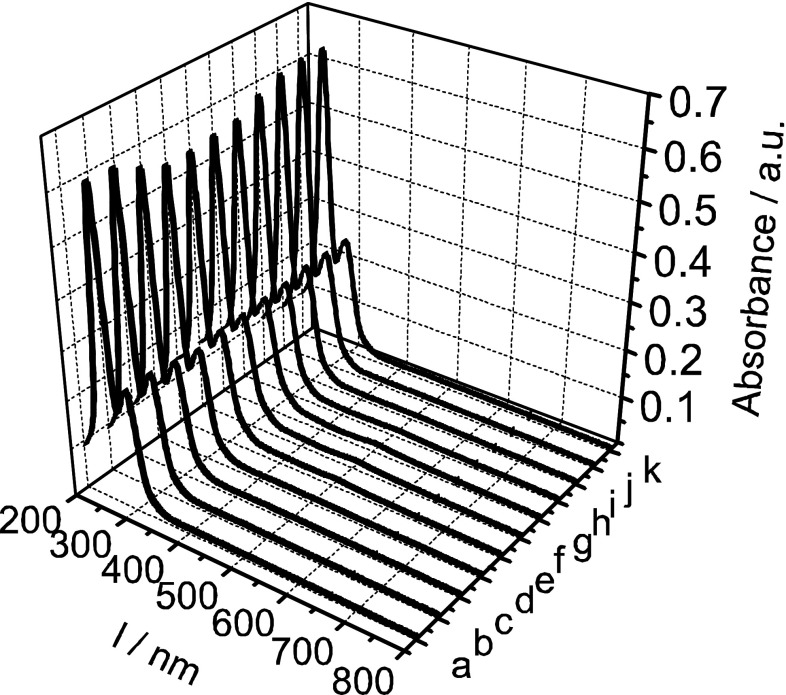
UV–Vis spectra recorded during cyclic voltammogram experiment in 0.5 mM KTcO_4_ + 0.5 M H_2_SO_4_

To confirm the conclusion on the electrochemical behaviour of technetium species in the acidic media, the spectroscopic signals were recorded simultaneously with the cyclic voltammograms. Figure 2 presents the UV–Vis spectra recorded during cyclic voltammetry of pertechnetate ions in 0.5 M H_2_SO_4_. No significant decrease of the TcO_4_^−^ ions concentration is observed for this acid concentration, as follows from an analysis of absorption bands at 244 and 288 nm. The obtained results are consistent with simultaneously recorded electrochemical data where only small noise current is recorded. For this acid concentration the discussed process is electrochemically irreversible on the carbon electrodes.

**Fig. 3:**
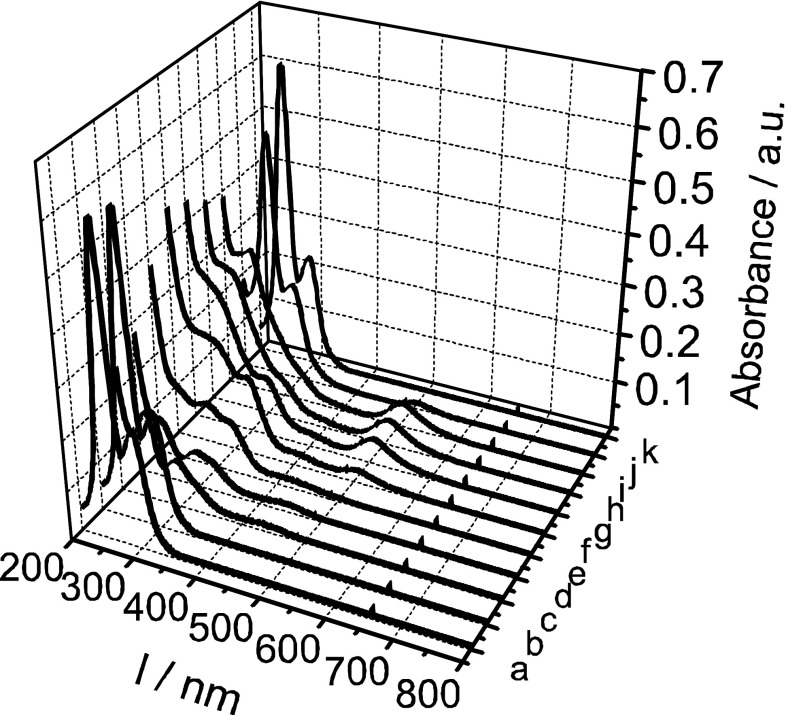
UV–Vis spectra recorded during cyclic voltammogram experiment in 0.5 mM KTcO_4_ + 4 M H_2_SO_4_

Totally different UV–Vis spectra are observed during the electroreduction of pertechnetate ions in much more concentrated acidic solutions (Fig. 3). In 4 M H_2_SO_4_, the technetium species with oxidation states lower than +VII are observed. The spectra reveal formation of a weak wave with a maximum in the range of 420–450 nm in the first step of the discussed process. At the same time a significant decrease of the concentration of the pertechnetate ions (*λ*_max_ = 244, 288 nm) is observed. For Mn(III) ions in the sulfuric acid solutions [20] a band with a maximum at 476 nm is observed. Generally, despite that the fact that the technetium is located in the same as the manganese group of periodic table, the absorption band for Tc should be shifted to a shorter wavelength as compared to Mn with the same ionic form and in the same medium [25]. Technetium(III) in his high spin aqueous complex, Tc(H_2_O)_6_^3+^, should have [Kr] 4d^4^ electronic configuration and from this reason the d–d transition should be allowed and respective bands in UV–Vis spectra should be observed. Such tendency has been observed in our experimental cell. The obtained results suggest that the wave with the maximum near 440 nm can be attributed to a technetium (III) ionic form. At higher potentials (0.7 V, see: UV–Vis spectrum for point “e” in Fig. 3) the observed wave shifts to shorter wavelengths. Based on the literature data [8] we suggest that this behaviour can indicate generation of soluble TcO^2+^ ions. At potentials higher than 0.8 V a band with a maximum at 502 nm is recorded. This band indicates that during the electrooxidation of the technetium with oxidation states lower than +IV the dimeric structures of Tc(III, IV) are generated.

**Fig. 4:**
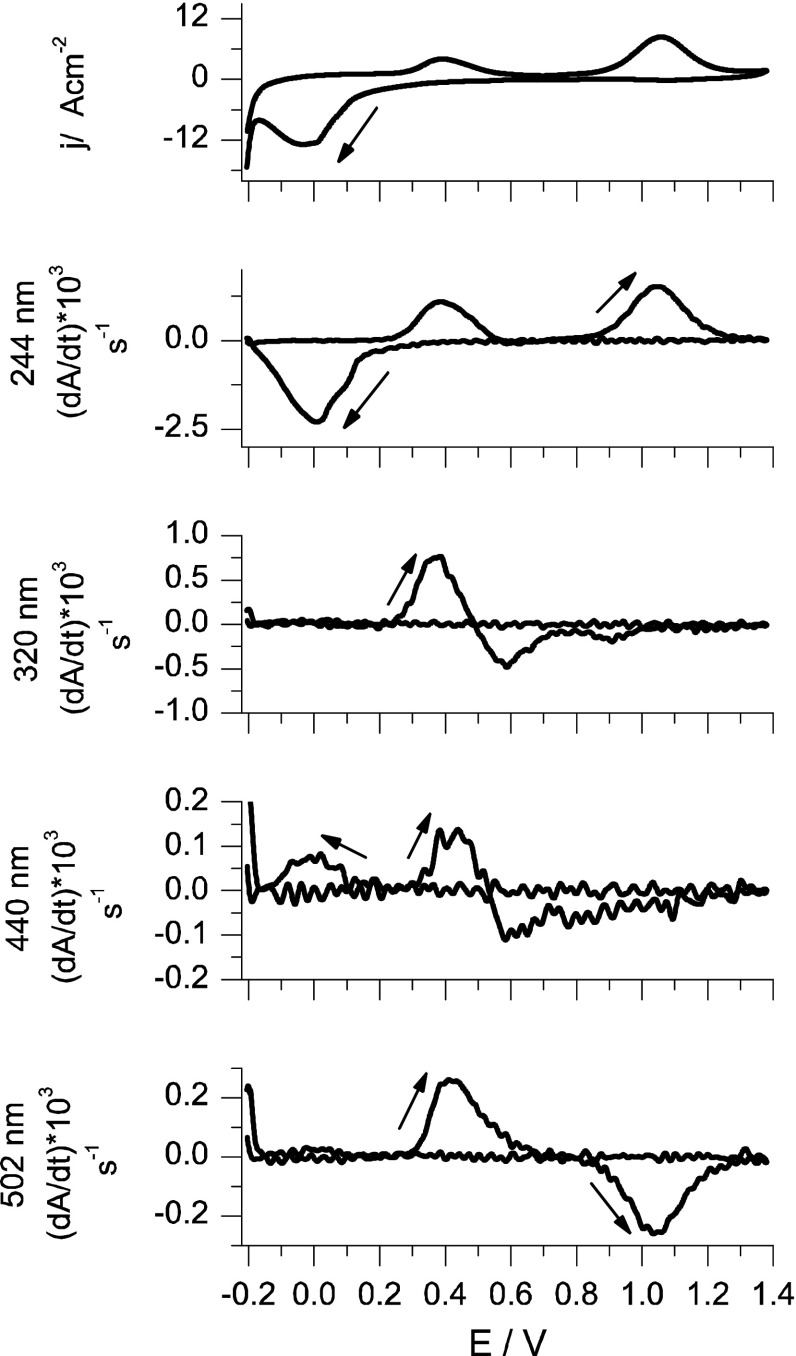
Cyclic voltammograms and voltabsorbommograms for wavelengths 244, 320, 440 and 504 nm of a OTTL-RVC electrode in 4 M H_2_SO_4_ + 0.5 mM KTcO_4_, v = 1 m V/s,* E*_start _= 0.6 V

Figure 4 presents a cyclic voltammogram recorded at scan rate of 1 mV/s and cyclic voltabsorbommograms calculated for the same scan rate for 244(TcO_4_^−^), 320 (Tc(IV)), 440 (Tc(III)), and 502 ([Tc(μ-O)_2_Tc]^3+/4+^) nm for a RVC-OTTL electrode in 4 M H_2_SO_4_. Electroreduction of pertechnetate ions in acidic media leads to formation of Tc(III) species, probably in the form of Tc^3+^ or TcO^+^. These species are responsible for formation of a positive” wave observed at potentials lower than 0.2 V on voltabsorbommogram calculated for the wavelength of 440 nm. No waves characteristic for technetium(III,IV) are observed in the same potentials range. One may suggest that these observations indicate generation of technetium(III) ionic forms as major products of perechnetate ions electroreduction in strongly acidic media. However, it is quite likely that before or after the generation of Tc(III) ions also other technetium species with higher than +III oxidation states are generated.

An anodic wave observed at cyclic voltammograms at potentials higher than 0.2 V is attributed to the electrooxidation of TcO^+^ or Tc^3+^ ions to technetium(III,IV) soluble species, e.g. [Tc(μ-O)_2_Tc]^3+/4+^. At these potentials a positive wave on voltabsorbommogram calculated for 502 nm) appears. These species are later electrooxidized to pertechnatate ions at relatively high potentials (higher than 0.9 V).

The band at 320 nm corresponds to a molecular electronic transition of polymeric Tc(IV) species [5]. For this wavelength the negative wave appears at much lower potentials (start at 0.5 V) than for dimeric structure of Tc(III, IV) (start at 0.8 V). This observation suggests that the wave at 320 nm is related not to dimeric structure of Tc(III, IV) but should be linked with other Tc(IV) species. [Tc(μ-O)_2_Tc]^3+/4+^ species generate much more intense negative wave at potentials higher than 0.8 V. Observed decrease of the absorbance at 502 nm (what is related with the “negative” wave on cyclic voltabsommograms) at potentials higher than 0.8 V indicates electrooxidation of these ions to TcO_4_^−^. Generation of pertechnetate ions in discussed range of potentials leads to an increase in the intensities of TcO_4_^−^ characteristic band (244 nm) and “positive” waves on cyclic voltabsorbommograms (at 0.4 and 1.0 V). In fact, the absorbance band at 320 nm attributed to Tc(IV) species should also increase simultaneously but this wavelength can be also attributed to Tc(VII) species. Therefore, a weak spectroscopic (0.8–1.0 V) signal is detected as a result of a superposition of waves for TcO_4_^−^ and Tc(IV).

**Fig. 5:**
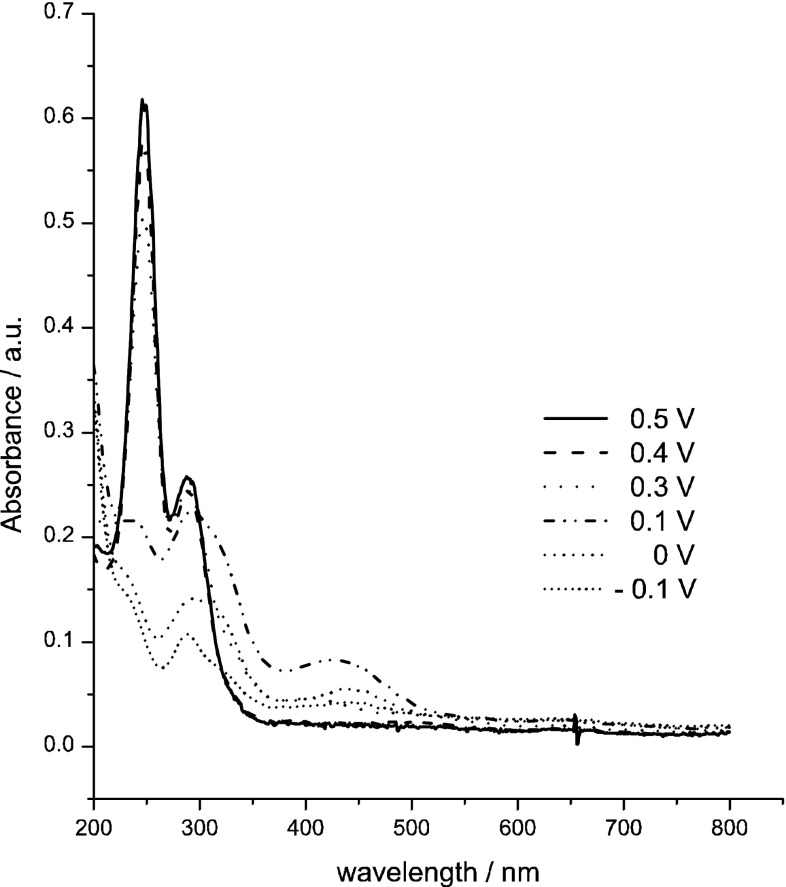
UV–Vis spectra recorded during chronoamperometric experiment in 0.5 mM KTcO_4_ + 4 M H_2_SO_4_. Applied potential program (1 min. for each step): 0.5 V → 1.1 V → 0.4 V → 1.1 V → 0.3 V → 1.1 V → 0.1 V → 1.1 V → 0 V → 1.1 V → −0.1 V

**Fig. 6:**
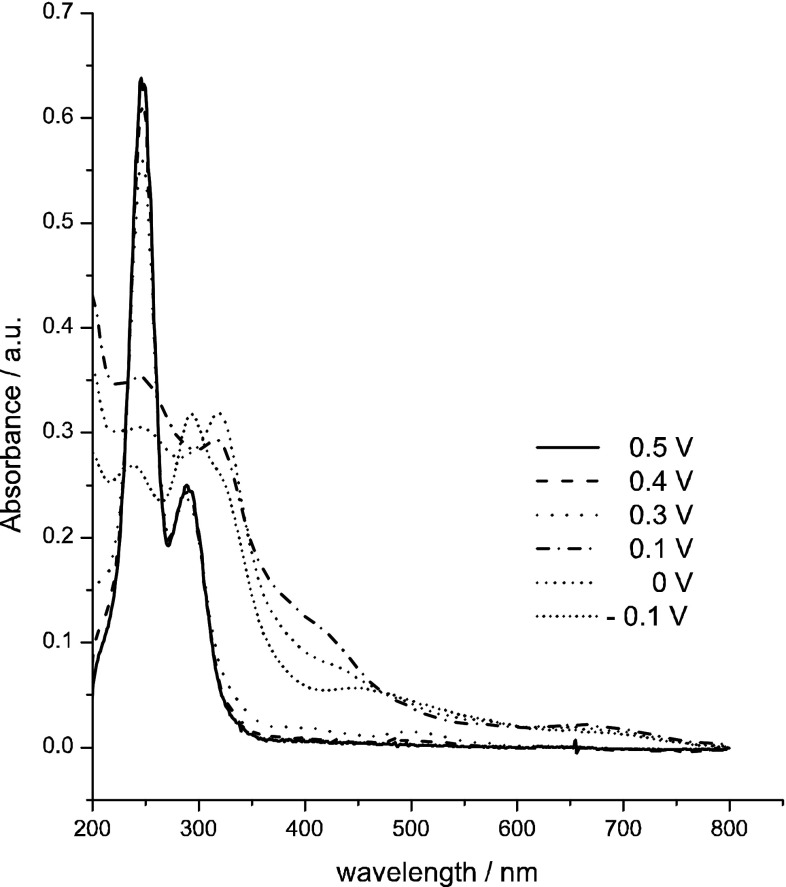
UV–Vis spectra recorded during chronoamperometric experiment in 0.5 mM·KTcO_4_ + 4 M·H_2_SO_4_. Applied potential program (1 min. for each step): 0.5 V → 0.4 V → 0.3 V → 0.1 V → 0 V → −0.1 V

Figures 5 and 6 present UV–Vis spectra recorded during chronoamperometric reduction of pertechnetate ions in 4 M H_2_SO_4_. Two procedures were applied:

- In the first procedure the potential was changed between fixed upper limit of 1.1 V and various lower limits in the range from 0.5 to −0.1 V with 0.1 V steps and with duration of 1 min., i.e. 0.5 V → 1.1 V → 0.4 V → 1.1 V → 0.3 V → 1.1 V → etc. (“direct” electroreduction of TcO_4_^−^, Fig. 5).
- The second procedure utilised stepwise changes of potential in cathodic direction with 0.1 V steps with duration of 1 min., i.e. 0.5 V → 0.4 V → 0.3 V → etc. (Fig. 6).

A comparison of Figs. 5 and 6 indicates that for the same value of reduction potential (e.g. 300 mV for both Figs. 5, 6) different reduced Tc species are generated in both procedures. “Direct” electroreduction of TcO_4_^−^ (Fig. 5) leads to generation of soluble technetium species characterised spectroscopically by the band at 440 nm. Stepwise electroreduction of pertechnetate ions in the second procedure leads to formation of bands at 320 and 248 nm which indicate existence of soluble structures of Tc(IV) and TcO_4_^−^. A similar UV–Vis spectrum (obtained in the solution with pH 0 was interpreted by Vongsouthi et al. [5] as a result of formation of a mixture of 20 % TcO_4_^−^ and 40 % Tc(IV, dimer) species. Further electroreduction of these species (Fig. 6,* E* = 0.3 V) leads to formation of technetium species characterised spectroscopically by bands at 244, 288 and 440 nm, characteristic for pertechnetate ions and for postulated by us Tc(III) soluble species. Our results are partially inconsistent with the results reported by Vongsouthi et al. [5] which indicated that Tc(III) species should not be detected spectroscopically in UV–Vis range. However, the acidity of the solutions used in our experiments (4 M·H_2_SO_4_) was much higher than in experiments performed by Vongsouthi et al. (pH 0) and this factor may influence the solubility of Tc(III) species. According to the recent literature data [4], formation of technetium (V) during the electroreduction of pertechnetate ions should be considered (Fig. 6, E = 0.1 V). At this value of potential a weak wave with the maximum centred at 650–700 nm is observed on UV–Vis spectrum. Poineau et al. [4] indicated that the molar absorption coefficient for technetium(V) for this band is low (*ε* = 34 M^−1^ cm^−1^). It is also puzzling that they reported generation of TcO^3+^ only in 13 M H_2_SO_4_ and not in 6 M H_2_SO_4_. In our experiments concentration of sulphuric acid was even lower (4 M H_2_SO_4_). From this reason it is also possible that the absorbance increase observed at 600–700 nm could be linked to hydrated technetium(III) oxohydroxides(?).

**Fig. 7:**
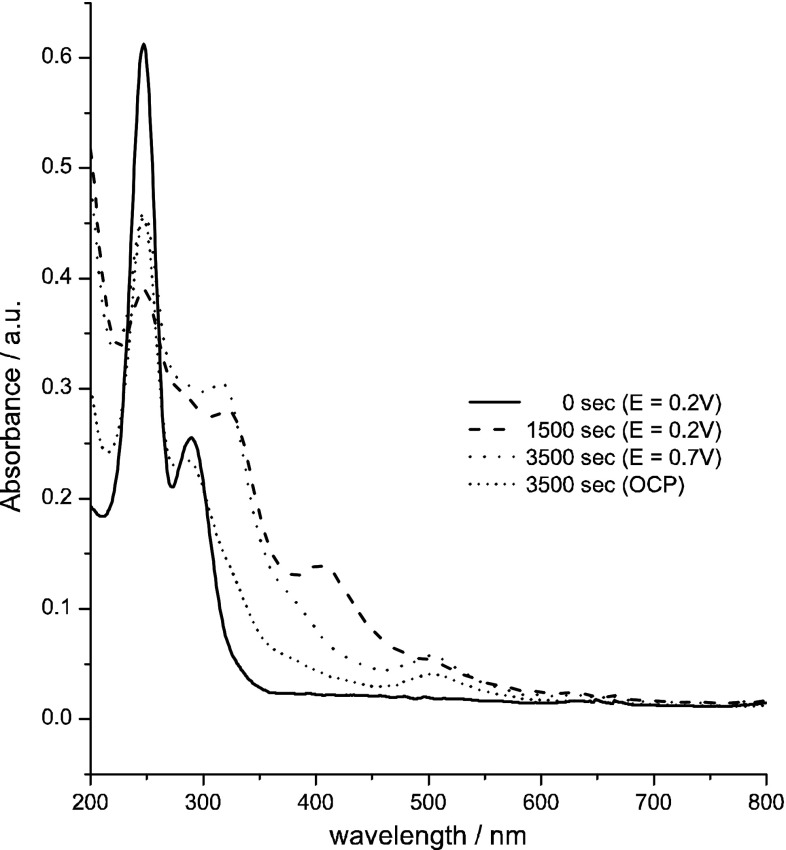
UV–Vis spectra recorded during chronoamperometric experiment and after them (at open circuit potential) in 0.5 mM KTcO_4_ + 4 M H_2_SO_4_

An additional chronoamperometric experiments were applied to determine the stability of selected technetium species in 4 M H_2_SO_4_. In Fig. 7 are presented spectra recorded during and after reduction of TcO_4_^−^ at applied potentials of 0 and 0.5 V and under open circuit conditions. At potential of 0 V the technetium(III) and (IV) forms are generated. The wave characteristic for TcO^2+^ with the maximum at 400 nm is misshapen due to overlapping with the wave characteristic for Tc(III) ions. Additionally, a weak shoulder at 500 nm indicates the appearance of [Tc(μ-O)_2_Tc]^3+/4+^ ions in the solution. At 0.5 V, a significant decrease of the absorbance in the UV–Vis range (from 370 to 470 nm) and an increase of the absorbance at 248 nm are observed. Such behaviour indicates that at this potential TcO^2+^ and Tc(III) forms are electrooxidized to pertechnetate ions. After 2,500 s at open circuit potentials, the dimeric structures of technetium(IV) are still present in the solution. However, at open circuit the intensity of the wave connected with these forms decreased about twice in comparison to conditions when the electrode was polarized at potential 0.5 V.

## Conclusions

During the electroreduction of pertechnetate ions in acidic media the technetium soluble species with +III and +IV oxidation states are generated. The spectroelectrochemical thin layer technique allowed the identification of particular technetium forms. Tc(III) ions can be spectroscopically characterized by the band with the maximum near 440 nm. These species can be easy oxidized to technetium(IV) existing in the solution as simple TcO^2+^ ions and dimeric forms of Tc(III,IV), [Tc(μ-O)_2_Tc]^3+/4+^. Chronoamperometric experiments clearly show that monooxotechnetium(IV) ions are much easier oxidized to pertechnetate ions than dimeric structures of Tc(III,IV).

## References

[1] Hu Q, Atwood DA (2010). Radionuclides in the environment.

[2] Loveland WD, Morrissey DJ, Seaborg GT (2006). Modern nuclear chemistry.

[3] Hu Q-H, Weng J-Q, Wang J-S (2010). J Environ Radioact.

[4] Poineau F, Weck PF, Burton-Pye BP, Denden I, Kim E, Kerlin W, German KE, Fattahi M, Francesconi LC, Sattelberger AP, Czerwinski KR (2013). Dalton Trans.

[5] Vongsouthi N, Fattahi M, Grambow B (2006–2008) Subatech UMR 6457 Laboratoire de Physique Subatomique et des Technologies Associées, Scientific Report http://www-subatech.in2p3.fr/Documents/SUBATECH2009.pdf

[6] Mausolf E, Poineau F, Droessler J, Czerwinski KR (2011). J Radioanal Nucl Chem.

[7] Vichot L, Fattahi M, Musikas C, Grambow B (2003). Radiochim Acta.

[8] Tumanova DN, German KE, Peretrukhin VF, Yu Tsivadze (2008). A Dokl Phys Chem.

[9] Heineman WR, Hawkridge FM, Blount HN (1984). Electroanalytical chemistry.

[10] Hurst RW, Heineman WR, Deutsch E (1981). Inorg Chem.

[11] Paquette J, Lawrence WE (1985). Can J Chem.

[12] Huber EW, Heineman WR, Deutsch E (1987). Inorg Chem.

[13] Chotkowski M, Czerwiński A (2012). Electrochim Acta.

[14] Rulfs L, Pacer RA (1967). Hirsch RF.

[15] Bancroft EE, Sidwell JS, Blount HN (1981). Anal Chem.

[16] Norwell VE, Mamantov G (1977). Anal Chem.

[17] Zamponi S, Czerwiński A, Gambini G, Marassi R (1992). J Electroanal Chem.

[18] Zamponi S, DiMarino M, Czerwiński A, Marassi R (1988). J Electroanal Chem.

[19] Zamponi S, Czerwiński A, Marassi R (1989). J Electroanal Chem.

[20] Chotkowski M, Rogulski Z, Czerwiński A (2011). J Electroanal Chem.

[21] Lawson BL, Scheifers SM, Pinkerton TC (1984). J Electroanal Chem.

[22] Grassi J, Devynck J, Trèmillon B (1979). Anal Chim Acta.

[23] Maslennikov AG, Courson O, Perettroukhine VE, David F, Masson M (1997). Radiochim Acta.

[24] Rard JA (1999). Chemical thermodynamics of technetium.

[25] Kettle SFA, Langmuir M (1996). Physical inorganic chemistry. A coordination chemistry approach.

